# Mechanistic Insights Into Overloading‐Induced Terminal Differentiation of TMJ Condylar Cartilage at the Single Cell Level

**DOI:** 10.1002/smmd.70011

**Published:** 2025-07-30

**Authors:** Dian Zhou, Yiling Jiang, Yingcui Li, Huanyu Zeng, Xinchun Li, Yufang He, Xin Wang, Yiteng Liang, Vojtech Parizek, Ousheng Liu, Zhangui Tang, Yueying Zhou

**Affiliations:** ^1^ Hunan Key Laboratory of Oral Health Research Hunan 3D Printing Engineering Research Center of Oral Care Hunan Clinical Research Center of Oral Major Diseases and Oral Health Xiangya Stomatological Hospital Xiangya School of Stomatology Central South University Changsha China; ^2^ Department of Biology University of Hartford West Hartford Connecticut USA; ^3^ Hradec Králové University Hospital Hradec Králové Czech Republic

**Keywords:** Acvr1b, condylar cartilage, single cell, temporomandibular joint, terminal differentiation

## Abstract

The incidence of temporomandibular joint (TMJ) degeneration has been steadily increasing, with overloading identified as a major risk factor. This condition often leads to condylar cartilage degeneration, significantly affecting patients' quality of life; however, the molecular mechanisms underlying this process remain poorly understood, and effective treatments are still lacking. We utilized single‐nucleus RNA sequencing to analyze the condylar cartilage in an overloading mouse model. This approach enabled the identification of 11 distinct cell types within the condylar chondrocytes. Through the application of pseudotime trajectory Analysis and cellchat analyses, we identified the key gene Acvr1b and its associated signaling pathway, which are crucial for regulating the terminal differentiation of condylar chondrocytes. This study utilized single‐nucleus RNA sequencing and in vitro validation to investigate the role of Acvr1b in TMJ cartilage degeneration under overloading stress. Our findings reveal key pathways involved in chondrocyte differentiation, providing a theoretical basis for the development of targeted therapeutic interventions.

## Summary

- Overloading shifts chondrocytes from proliferative to hypertrophic states, accelerating terminal differentiation.
- Identified Acvr1b as a key regulator of chondrocyte terminal differentiation in TMJ degeneration via TGF‐β/Smad3 signaling.
- Targeting Acvr1b reduces terminal differentiation markers, offering a potential therapeutic strategy.

## Graphical abstract

Using single‐nucleus RNA sequencing, we identified Acvr1b as a key regulator of chondrocyte terminal differentiation via the TGF‐β pathway under mechanical overloading—a major driver of temporomandibular joint (TMJ) degeneration. Our work provides the first evidence of Acvr1b′s role in TMJ cartilage pathology, offering a potential therapeutic target to mitigate degeneration.

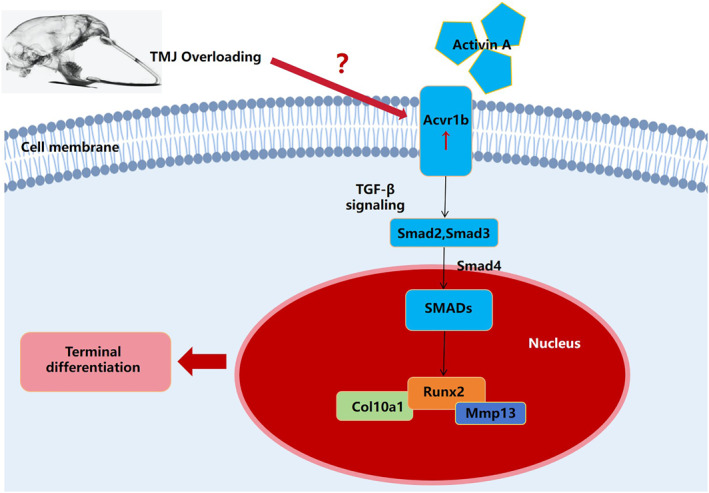

## 1 Introduction

The temporomandibular joint (TMJ) is a complicated weight‐bearing joint that is essential for chewing, speaking, and maintaining facial features [1]. There is mounting clinical evidence that the rising incidence of TMJ degeneration, especially in younger populations, is caused by elements including psychological stress and aberrant occlusal forces [2, 3]. A well‐established body of evidence suggests that mechanical overloading disrupts articular cartilage homeostasis, leading to degenerative changes in cartilage and subchondral bone resorption [4, 5, 6, 7]. In the TMJ, this degenerative process manifests clinically through enhanced terminal differentiation of condylar chondrocytes, accelerated cellular maturation, and aberrant chondrocyte terminal differentiation [8] and is typified by inflammatory reactions, reduced mechanical cushioning, and the erosion of cartilage layers [9]. Joint discomfort, limited mobility, and substantial declines in quality of life are frequently the results of these alterations [2].

Current therapies generally rely on conservative measures such as heat therapy, analgesics, occlusal pad application, and intra‐articular injections, which provide temporary relief but do not address the underlying degradation of TMJ cartilage [10]. Total joint replacement, the last resort in severe instances, has limits because artificial joints cannot duplicate the load‐bearing and regenerating qualities of native cartilage [11]. As a result, there is an urgent need to investigate non‐invasive, effective therapeutics targeting the molecular pathways behind TMJ degeneration.

New research suggests that overloading causes TMJ degeneration through processes such as endoplasmic reticulum stress, which promotes chondrocyte death and autophagy, as well as parathyroid hormone receptor‐1‐mediated differentiation [12, 13]. However, these processes have a limited regenerating capacity for injured cartilage. Identifying progenitor cells capable of cartilage repair and understanding the processes that control overloading‐induced differentiation are critical steps toward designing viable therapeutics. Our preliminary experiments have demonstrated that Activin A is implicated in the terminal differentiation of condylar cartilage under mechanical overloading. While ACTIVIN receptors are critically involved in bone metabolism, with Acvr1b playing a pivotal regulatory role in cellular differentiation across multiple lineages [14, 15, 16], its specific role in regulating terminal differentiation of TMJ condylar cartilage remains uncharacterized. This work aims to understand the involvement of Acvr1b in these processes, including its connection with the TGF‐β signaling pathway.

## 2 Results

### 2.1 Overloading Leads to an Increase in the Proportion of the Chondrocyte Cluster

To investigate the mechanisms underlying TMJ degeneration caused by overloading, we developed a TMJ degeneration model using a mouse large‐mouth opening approach.

**FIGURE 1:**
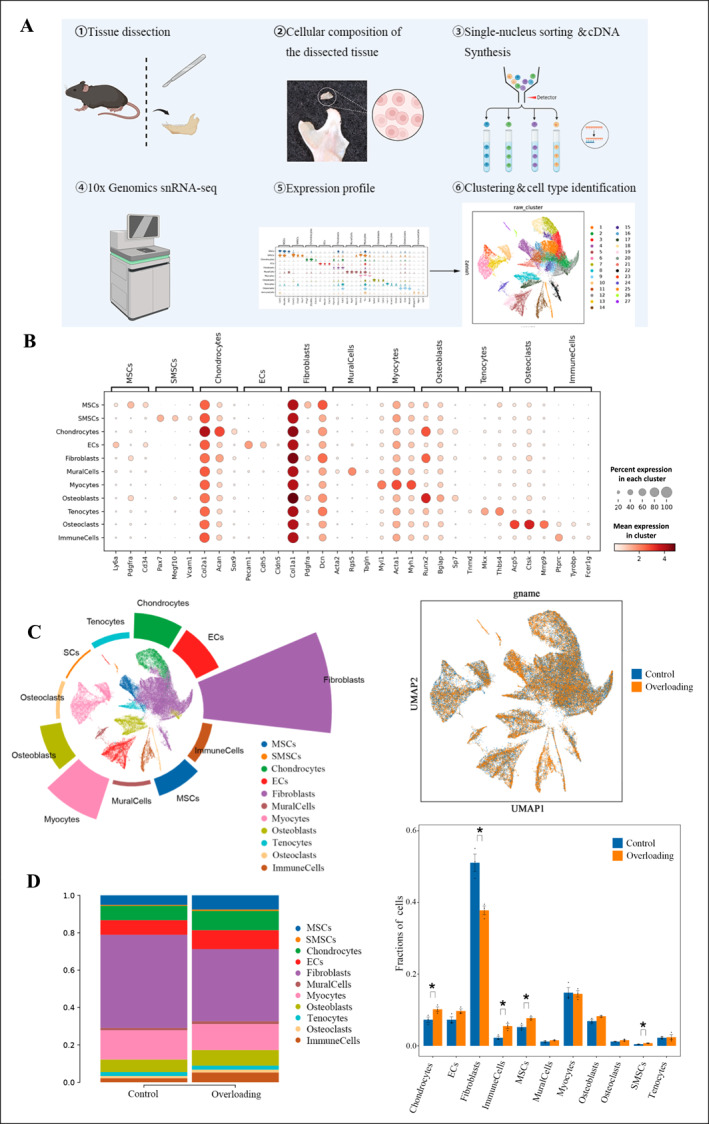
Overloading leads to an increase in proportion of the chondrocyte cluster: (A) Schematic representation of the single‐nucleus RNA sequencing protocol. (B) Top three differentially expressed genes across 11 distinct cell populations. (C) Comparative UMAP visualizations of 11 cell types between the Overloading group and the control group. (D) Graphical representation of proportional changes and statistical significance for 11 cell types between the Overloading group and the control group. All data are shown as means ± SEM. *, *p* < 0.05.

In this study, we constructed a comprehensive cell atlas based on six samples, analyzing a total of 59,734 cells—32,845 from the control group and 26,889 from the overloading group. To explore cellular heterogeneity, we utilized Scanpy for cell clustering based on gene expression profiles, followed by dimensionality reduction using t‐distributed stochastic neighbor embedding (t‐SNE) and uniform manifold approximation and projection (UMAP) for visualization. This analysis identified 27 distinct unsupervised clusters (Figure 1A).

We identified 11 cell types through differential gene expression analysis, including mesenchymal stem cells (MSCs), skeletal muscle satellite cells (SMSCs), chondrocytes, endothelial cells (ECs), fibroblasts, mural cells, myocytes, osteoblasts, tenocytes, osteoclasts, and immune cells (Figure 1B,C). Notably, the proportions of chondrocytes (control: 7.68%, overloading: 10.31%), immune cells (control: 2.11%, overloading: 5.19%), MSCs (control: 5.15%, overloading: 7.62%), and SMSCs (control: 0.50%, overloading: 0.74%) were significantly higher in the overloading group. In contrast, the proportion of fibroblasts decreased significantly (control: 49.85%, overloading: 38.54%) (Figure 1D).

### 2.2 Overloading Promotes the Expression of Acvr1b

**FIGURE 2:**
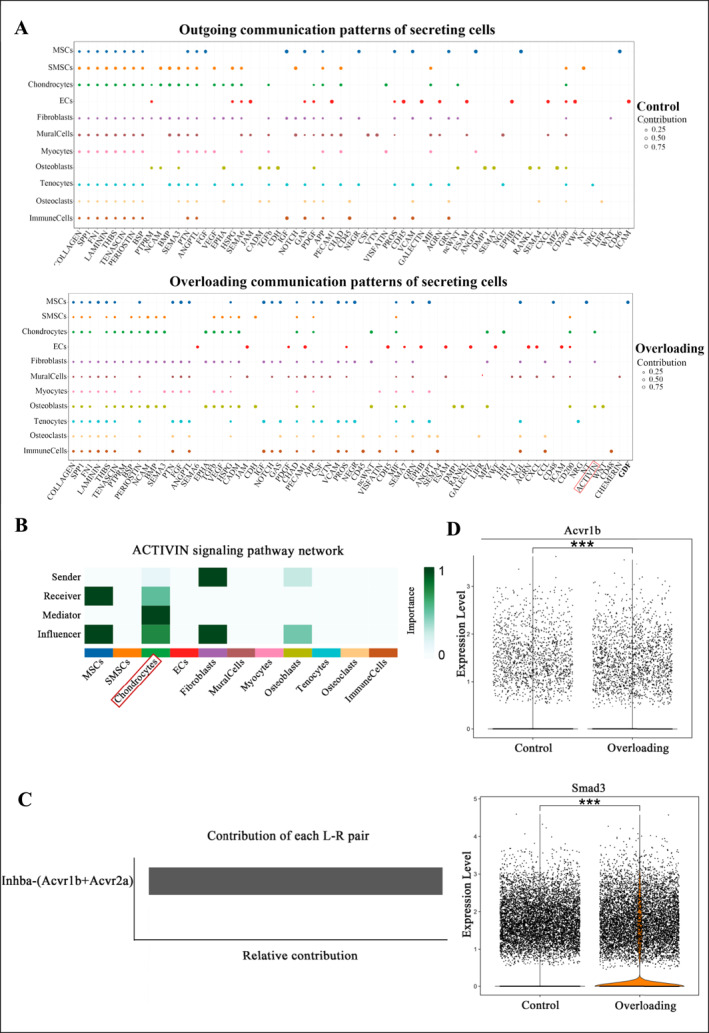
Overloading promotes the expression of Acvr1b: (A) Cellular communication dot plots comparing the Overloading group to the control group. (B) Network diagram illustrating the ACTIVIN signaling pathway. (C) Contribution plot of ligands to the ACTIVIN signaling pathway. (D) Expression of Acvr1b and Smad3 in the Overloading group versus the control group. All data are shown as means ± SEM. ***, *p* < 0.001.

To identify the key regulatory elements involved in mechanical overload, we conducted a comprehensive cell communication analysis across different cellular populations. Our results revealed a significant increase in ACTIVIN signaling interactions following mechanical overload (Figure 2A). Notably, chondrocytes, fibroblasts, and osteoclasts were identified as the primary signal‐emitting cells in this pathway, while chondrocytes and MSCs were the main signal‐receiving cells. Interestingly, ACTIVIN signaling was found to influence chondrocytes throughout their lifecycle (Figure 2B), underscoring its central role in regulating these cells under mechanical stress.

Within the ACTIVIN signaling pathway, Acvr1b and Acvr2a were the most prominent receptors, with Inhba being the most influential ligand (Figure 2C). Acvr1b is involved in a wide range of biological processes, including cellular differentiation, and exerts its effects mainly through the Smad signaling pathway during osteoblast differentiation. Specifically, Smad3 has been shown to promote chondrogenesis and maintain the chondrocyte phenotype by upregulating key genes involved in cartilage matrix synthesis, such as aggrecan and type II collagen. Our comparative analysis of Acvr1b and its downstream effector Smad3 in both experimental groups showed increased expression following mechanical overload (Figure 2D), suggesting that Acvr1b may be a critical target for modulating degenerative changes in the TMJ. Building on these findings, future studies will focus on further understanding the interaction between Acvr1b and chondrocytes.

### 2.3 Overloading Promotes the Terminal Differentiation of Chondrocyte Subclusters One to Three

Given that overloading was the primary factor in this study, and that chondrocytes are the main stress‐sensing cells in condylar cartilage, we focused on the chondrocyte cluster. Chondrocytes vary in cell density, size, and the extent of matrix calcification. Based on these differences, chondrocytes can be classified into four layers from superficial to deep: the fibrous layer, proliferative zone, chondrocyte layer, and hypertrophic layer, with each layer exhibiting varying degrees of cell differentiation.

**FIGURE 3:**
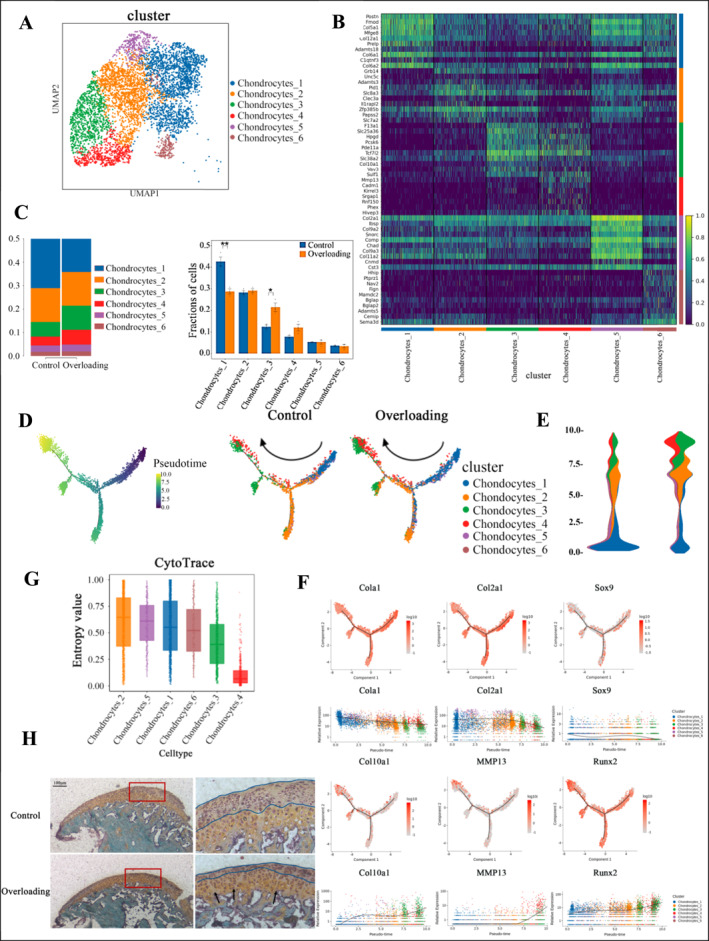
Overloading promotes the terminal differentiation of chondrocyte subclusters 1 to subclusters 3: (A) UMAP plots of chondrocyte subpopulations. (B) Heatmap of representative genes for each chondrocyte subpopulation. (C) Graphs of proportional changes and statistical significance for six chondrocyte subpopulations between the Overloading group and the control group. (D) Pseudotime trajectory plots for the Overloading group and the control group. (E) Pseudotime density variation plots for the Overloading group and the control group. (F) Pseudotime expression profiles of representative genes in each layer of condylar cartilage. (G) CytoTrace analysis for each chondrocyte subpopulation. (H) Safranin O (SO) staining for the Overloading group and the control group. All data are shown as means ± SEM. *, *p* < 0.05; **, *p* < 0.01.

To better understand how overloading affects chondrocyte differentiation, we further subdivided the chondrocyte cluster into six distinct subclusters based on differential gene expression (Figure 3A,B).

Comparing the proportions of each chondrocyte subcluster between the control and overloading groups, we found that the number of chondrocyte‐1 cells significantly decreased under overloading (control: 42.12%, experimental: 28.33%), while the proportion of chondrocyte‐3 cells increased (control: 12.48%, experimental: 20.58%), with statistically significant differences (*p* < 0.05) (Figure 3C).

To explore the differentiation relationships between chondrocyte subclusters, we conducted pseudotime analysis, categorizing all chondrocytes into five states based on varying degrees of differentiation. A pseudotime trajectory plot was generated, where darker colors represent the starting point and lighter colors indicate cells at a greater distance from the origin (Figure 3D). This analysis revealed that chondrocyte‐1 cells differentiate into chondrocyte‐3 and chondrocyte‐4 cells.

Furthermore, we observed that overloading caused a decrease in the density of chondrocyte‐1 cells, while the densities of chondrocyte‐3 and chondrocyte‐4 cells increased (Figure 3E).

To further characterize the differentiation status of each chondrocyte subcluster, we selected representative genes associated with different stages of chondrocyte differentiation: Col1a1, Col2a1, Sox9, Col10a1, Mmp13, and Runx2. The expression of these genes was analyzed in relation to pseudotime, with the *x*‐axis representing pseudotime from low to high values, and the *y*‐axis representing gene expression levels. Different cell types were color‐coded for clarity. The results showed that chondrocyte‐1 predominantly expresses genes characteristic of the proliferative zone, such as Col1a1, Col2a1, and Sox9. As pseudotime progresses, chondrocyte‐3 cells highly express genes typical of the hypertrophic layer, including Col10a1, Mmp13, and Runx2. This suggests that chondrocyte‐1 cells are less differentiated, whereas chondrocyte‐3 cells are more differentiated. Additionally, chondrocyte‐2, located between chondrocyte‐1 and chondrocyte‐3, likely represents a transitional state in the differentiation process (Figure 3F).

We then performed CytoTRACE analysis to assess the stemness and differentiation potential of each subcluster by calculating entropy scores. The *x*‐axis represents the different chondrocyte subtypes, and the *y*‐axis represents the corresponding entropy scores. A higher entropy score indicates a higher degree of stemness and differentiation potential. The analysis revealed that chondrocyte‐1 had a higher entropy score, indicating a greater stemness and differentiation potential, while chondrocyte‐3 and chondrocyte‐4 had lower entropy scores, suggesting that differentiation is more complete in these subclusters (Figure 3G).

These findings suggest that chondrocyte‐1 cells are primarily proliferative layer cells, whereas chondrocyte‐3 and chondrocyte‐4 cells represent the more differentiated hypertrophic layer cells. Based on these observations, we deduced that under overloading, the proliferative layer of condylar cartilage becomes thinner, while the hypertrophic layer becomes thicker. This is consistent with the histological staining results in mice (Figure 3H), which also show a shift in differentiation from proliferative to hypertrophic cells. Therefore, identifying the key pathways that promote terminal differentiation is crucial for regulating chondrocyte differentiation and intervening in cartilage degeneration.

Additionally, we performed Safranin O‐fast green staining on sagittal sections of the TMJ to observe changes in different chondrocyte types. The condylar cartilage appeared red, whereas the subchondral bone was stained green. Our results showed that under overloading, the proliferative zone of condylar cartilage was thinner (indicated by green lines), whereas the pre‐hypertrophic and hypertrophic cell layers were thicker (highlighted by orange‐red staining). We also observed the appearance of acellular clefts in the cartilage layer (marked by black arrows).

### 2.4 Acvr1b May be a Key Gene Regulating Chondrocyte Terminal Differentiation

Through cell communication analysis, we identified the most critical cytokines involved in this process. We assessed the strength of cell‐to‐cell interactions among the subclusters, where the thickness of the lines indicates the interaction strength, and the color represents the ligand‐producing cell type.

**FIGURE 4:**
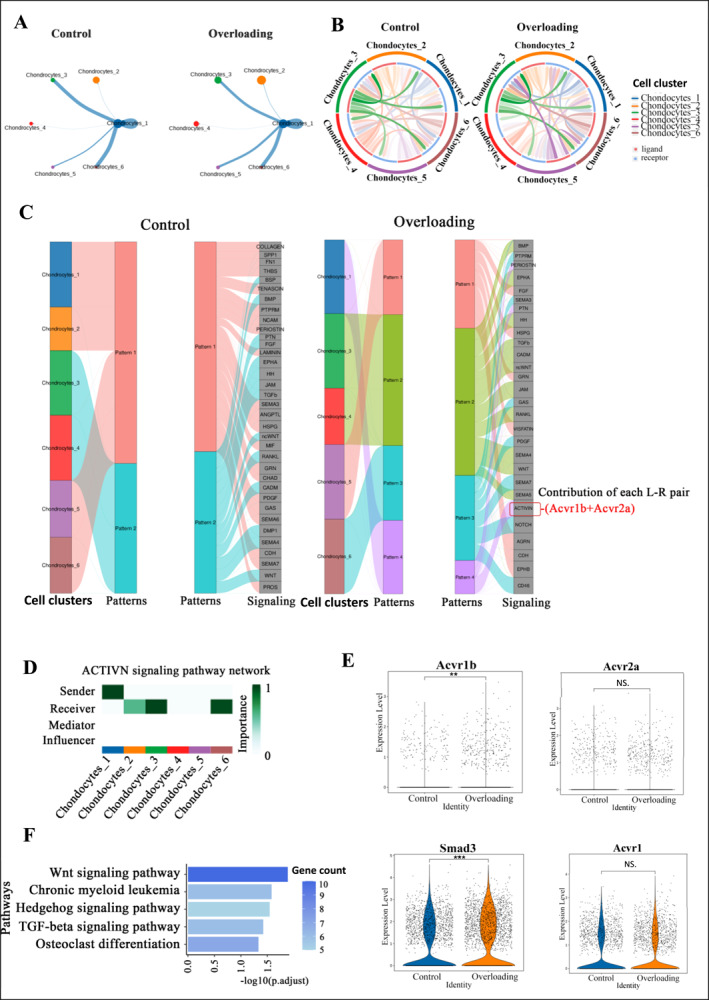
Acvr1b may be a key gene regulating chondrocyte terminal differentiation: (A) Cellular communication alterations in Chondrocyte Subpopulation 1 between the Overloading group and the control group. (B) Cellular interaction maps for each chondrocyte subpopulation within the Overloading group and the control group. (C) Cellular communication pattern diagrams for chondrocyte subpopulations in the Overloading group and the control group, with a focus on the specific expression of ACTIVIN and the identification of the highest contributing receptors, Acvr1b and Acvr2a, through ligand contribution analysis. (D) Network diagram delineating the ACTIVIN signaling pathway. (E) Expression profiles of Acvr1b, Acvr2a, Smad3, and Acvr1 in chondrocyte populations between the Overloading group and the control group. (F) KEGG analysis of signaling pathways upregulated following overloading. All data are shown as means ± SEM. *, *p* < 0.05; **, *p* < 0.01; ***, *p* < 0.001; NS., *p* > 0.05.

Previous studies suggest that the differentiation of chondrocyte‐1 into chondrocyte‐3 may represent the transition from the proliferative zone to the hypertrophic zone within condylar cartilage. To identify key genes regulating this process, our analysis showed that after overloading, interactions between chondrocyte‐1 and chondrocyte‐3, as well as between chondrocyte‐1 and other subclusters such as chondrocyte‐2, chondrocyte‐5, and chondrocyte‐6, were significantly enhanced, with the strongest interaction observed between chondrocyte‐1 and chondrocyte‐3 (Figure 4A).

The receptor‐ligand interaction diagram illustrates the different cell types on the outer circle, with the thickness of the arrows indicating the probability of communication. Thicker arrows represent stronger interactions. We found that under overloading, chondrocyte‐3 received a stronger signal from other chondrocyte subclusters (Figure 4B). These results suggest that under overloading, chondrocyte‐1 increases its output signals, and chondrocyte‐3 becomes the main recipient. This indicates that chondrocyte‐1 and chondrocyte‐3 are key subclusters involved in the overloading response.

Further analysis of the output signals from chondrocyte‐1 revealed a specific increase in the interaction with ACVIVIN (Figure 4C), indicating that ACVIVIN is specifically activated in condylar cartilage under mechanical overload. To identify the key genes involved, we analyzed the contribution of ligands and receptors throughout the signaling pathway. The most influential ligand was Inhba, while Acvr1b and Acvr2a were the most important receptors (Figure 4C). These findings suggest that Acvr1b and Acvr2a play a critical role in regulating the terminal differentiation of chondrocyte subclusters.

Analysis of this signaling pathway revealed that chondrocyte‐1 acts as a signal emitter, while chondrocyte‐2, chondrocyte‐3, and chondrocyte‐6 are signal recipients (Figure 4D). This suggests that, under overloading, chondrocyte‐1 transmits signals to more differentiated chondrocyte‐3 cells and intermediate chondrocyte‐2 cells.

To further investigate the roles of ACVIVIN, Acvr1b, and Acvr2a in cell communication, we examined their expression in the chondrocyte subclusters. The results showed that, under overloading, the expression of Acvr1b and its downstream effector Smad3 significantly increased (*p* < 0.05), while there was no significant difference in the expression of Acvr2a (*p* > 0.05) (Figure 4E). We also analyzed the expression of Acvr1a, the type I receptor for ACVIVIN, and found no significant difference between the two groups (*p* > 0.05). These results suggest that Acvr1b may be a key target for regulating chondrocyte terminal differentiation under overloading conditions. Furthermore, KEGG enrichment analysis of chondrocyte subclusters revealed upregulation of the TGF‐β signaling pathway after overloading (Figure 4F). Since ACVIVIN, a ligand for Acvr1b, is a member of the TGF‐β family, and both share the downstream factor Smad3, these findings indicate that Acvr1b may regulate the terminal differentiation of condylar cartilage through the TGF‐β signaling pathway.

### 2.5 Overloading Promotes the Expression of Acvr1b in Mice Condylar Cartilage Tissue

**FIGURE 5:**
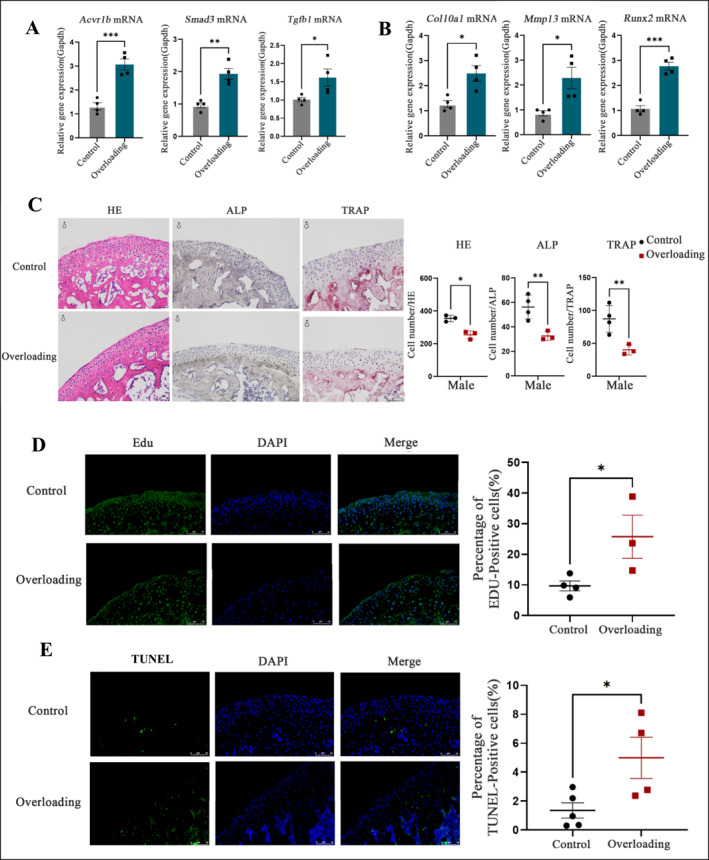
Overloading Promotes the Expression of Acvr1b in Mice Condylar Cartilage Tissue: (A) Expression profiles of Acvr1b‐related genes in male mice condylar cartilage tissue between the Overloading group and the control group. (B) Expression profiles of terminal differentiation‐associated genes in male mice condylar cartilage tissue between the Overloading group and the control group. (C) Hematoxylin and eosin (H&E) staining and cell counting of male mice condylar cartilage tissue in the Overloading group and the control group. (D) 5‐Ethynyl‐2'‐deoxyuridine (EdU) staining and statistical significance of male mice condylar cartilage tissue in the Overloading group and the control group. (E) Terminal deoxynucleotidyl transferase dUTP nick‐end labeling (TUNEL) staining and statistical significance of male mice condylar cartilage tissue in the Overloading group and the control group. All data are shown as means ± SEM. *, *p* < 0.05; **, *p* < 0.01; ***, *p* < 0.001.

To validate the expression of the key gene Acvr1b identified through single‐cell nuclear transcriptome sequencing, we first established a TMJ degeneration model in mice using a large‐mouth opening and force application device. After euthanizing the mice, we isolated condylar cartilage tissue and performed qPCR to measure gene expression. We found a significant increase in Acvr1b mRNA expression following overloading, along with increased expression of Tgfb1 mRNA and its downstream effector Smad3 mRNA, with statistical significance (*p* < 0.05) (Figure 5A). These results suggest that Acvr1b is linked to the TGF‐β signaling pathway, consistent with previous findings.

Next, we assessed the expression of genes related to terminal differentiation in condylar cartilage. qPCR analysis showed that overloading resulted in increased expression of Col10a1 mRNA, a marker for hypertrophic chondrocytes, as well as upregulation of Mmp13 mRNA and Runx2 mRNA, all of which were statistically significant (*p* < 0.05) (Figure 5B). These findings indicate that overloading promotes the expression of terminal differentiation genes in condylar cartilage.

Hematoxylin and eosin (HE) staining of sagittal TMJ sections from mice allowed us to observe cellular changes in condylar cartilage. The staining revealed that cell nuclei appeared blue, the cartilage matrix and calcium salts were stained dark blue, the cytoplasm ranged from pink to peach, and collagen fibers were pale pink. Our results showed that after overloading, male mice exhibited large acellular areas in the condylar cartilage, along with thinning of the cartilage layer and disorganized cell arrangement. Semi‐quantitative analysis of chondrocyte numbers from HE staining showed a significant reduction in cell count for male mice exposed to overloading, with a statistically significant difference (*p* < 0.05) (Figure 5C).

For alkaline phosphatase (ALP) staining, the cell nuclei were stained light blue, and ALP‐positive cells appeared brown to black. Male mice showed a decrease in ALP‐positive cells after overloading. Semi‐quantitative analysis revealed a significant reduction in the proportion of ALP‐positive cells in male mice (*p* < 0.05) (Figure 5C).

We also performed tartrate‐resistant acid phosphatase (TRAP) staining on TMJ sagittal sections. The cell nuclei were stained light blue, and TRAP‐positive osteoclasts exhibited a purplish‐red cytoplasm. After overloading, male mice showed a decrease in TRAP‐positive cell areas. Semi‐quantitative analysis revealed a significant reduction in TRAP‐positive cells for both genders, with male mice showing a more pronounced change (*p* < 0.05) (Figure 5C).

To assess the impact of overloading on chondrocyte proliferation, we administered EdU (10 μL/g) intraperitoneally to label proliferating cells 24 h prior to euthanasia. EdU staining marked proliferating cells in green, with nuclei stained blue. The results showed that overloading increased the proportion of proliferating cells. In the control group, EdU‐positive cells were predominantly localized to the proliferative zone of the condylar cartilage, whereas in the overloading group, they were more abundant in the hypertrophic zone. By calculating the percentage of EdU‐positive cells, we found a statistically significant increase in proliferating cells under overloading (*p* < 0.05) (Figure 5D). This suggests that, under normal loading, chondrocyte proliferation occurs mainly in the proliferative zone, whereas overloading shifts proliferation to the hypertrophic zone.

Finally, to examine chondrocyte apoptosis, we used the TUNEL method to detect apoptotic cells in the tissue samples, with DAPI staining used to visualize cell nuclei. The results revealed an increase in apoptotic cells at the junction between the hypertrophic cell layer and the subchondral bone under overloading. The ratio of TUNEL‐positive cells to total cells was significantly higher after overloading (*p* < 0.05) (Figure 5E). These findings indicate that overloading induces apoptosis in cells at the cartilage‐subchondral bone interface, contributing to bone tissue destruction.

### 2.6 Inhibiting Acvr1b Expression Restores Proliferation Capacity of Condylar Chondrocytes and Reduces the Expression of Terminal Differentiation‐Related Genes

**FIGURE 6:**
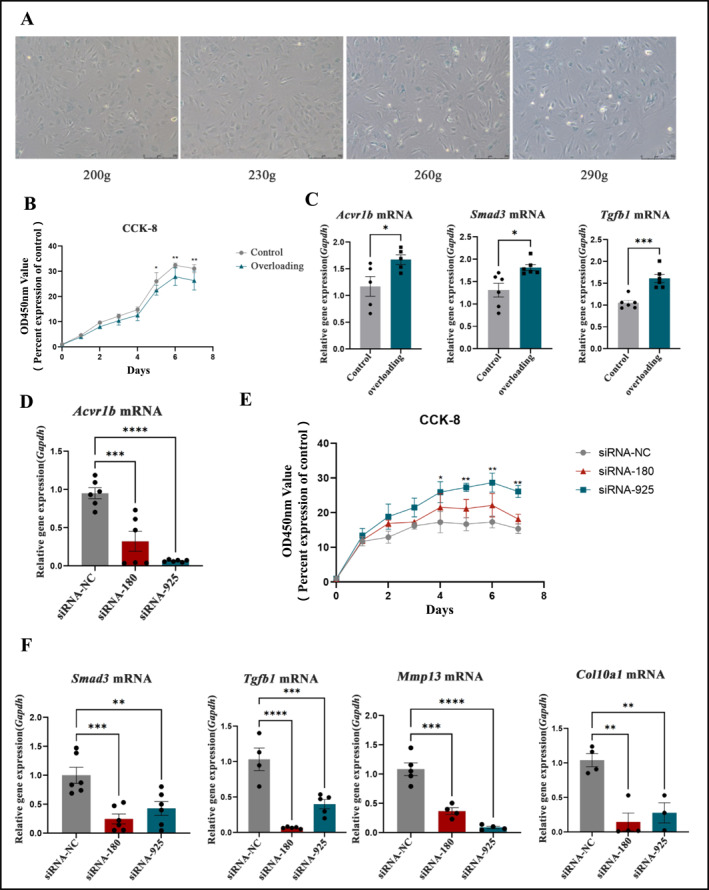
Inhibiting Acvr1b Expression Restores Proliferation Capacity of Condylar Chondrocytes and Reduces the Expression of Terminal Differentiation‐Related Genes: (A) Senescence‐associated *β*‐galactosidase (SA‐*β*‐Gal) staining patterns of condylar chondrocytes under varying centrifugal forces. (B) Proliferative status of condylar chondrocytes in the Overloading group compared to the control group. (C) Expression of Acvr1b, Smad3, and Tgfb1 in condylar chondrocytes between the Overloading group and the control group. (D) Gene expression profile of condylar chondrocytes following SiRNA Acvr1b treatment. (E) Proliferative capacity of condylar chondrocytes after SiRNA Acvr1b treatment. (F) Gene expression analysis of Smad3, Tgfb1, Mmp13, and Col10a1 in condylar chondrocytes treated with SiRNA Acvr1b. All data are shown as means ± SEM. *, *p* < 0.05; **, *p* < 0.01; ***, *p* < 0.001.

To examine the effects of varying centrifugal forces on condylar cartilage cells, we subjected the second‐generation chondrocytes to different centrifugal forces. At 200–230g, no significant changes in cell morphology were observed. However, as the centrifugal force increased, the size of the chondrocytes enlarged, the number of cell nuclei increased, cell boundaries became less distinct, and the number of cell protrusions increased. After staining the cells with SA‐β‐Gal, we observed that the number of positively stained cells (appearing blue) also increased with increasing centrifugal force, indicating that overloading accelerates cellular aging, with the most pronounced effect at 290g (Figure 6A). Based on these findings, subsequent experiments were conducted with the control group subjected to 200g centrifugal force, and the overloading group subjected to 290g.

To verify whether the in vitro cell overloading phenotype is consistent with the in vivo phenotype, we first assessed the growth curve of condylar cartilage cells using the CCK‐8 assay. The results showed that between the 120‐ and 168‐h time points, the absorbance of the control group was significantly higher than that of the overloading group, with a statistically significant difference (*p* < 0.05) (Figure 6B). This indicates that overloading reduces the growth rate of condylar cartilage cells, with the reduction becoming more pronounced over time.

Further qPCR analysis of condylar cartilage cells revealed a significant increase in Acvr1b mRNA expression following overloading, along with upregulation of Smad3 mRNA and Tgfb1 mRNA (*p* < 0.05) (Figure 6C). These results align with the expression changes observed in condylar cartilage tissue from overloading mice.

Given the increased expression of Acvr1b in condylar cartilage cells after overloading, we used siRNA to knock down Acvr1b expression and assessed the changes in gene expression by qPCR. The results showed that siRNA‐180 and siRNA‐925 effectively suppressed Acvr1b expression at the gene level (*p* < 0.05) (Figure 6D).

After confirming the successful knockdown of Acvr1b, we transfected the condylar cartilage cells in a 96‐well plate after applying overloading for 24 h. Since siRNA interference is transient, lasting only 96 h, we evaluated cell growth over 5 days, that is 120 h. The results showed that at the 96‐ to 120‐h time points, the absorbance of siRNA‐180 and siRNA‐925 was higher than that of the siRNA‐NC group, with siRNA‐925 showing a significant difference (*p* < 0.05). This indicates that the knockdown of Acvr1b promotes cell growth under overloading conditions (Figure 6E).

To validate the mechanism by which Acvr1b regulates chondrocyte terminal differentiation, we transfected condylar cartilage cells on a 24‐well plate after applying overloading for 24 h. Following 24 h of transfection, we examined the effects of siRNA‐180 and siRNA‐925 on the expression of Tgfb1 and Smad3. The results showed that the knockdown of Acvr1b led to a significant downregulation of both Tgfb1 and Smad3 expression (*p* < 0.05), indicating that Acvr1b plays a specific regulatory role in the TGF‐β signaling pathway. Additionally, we measured the expression of terminal differentiation‐related factors Mmp13 and Col10a1. The data showed that knocking down Acvr1b resulted in the downregulation of these genes (*p* < 0.05) (Figure 6F). This suggests that reducing Acvr1b expression in condylar cartilage inhibits terminal differentiation of chondrocytes.

## 3 Discussion

Our study established a mouse TMJ degeneration model under overloading [5] and provides the first single‐cell resolution evidence that overloading drives TMJ degeneration by accelerating chondrocyte terminal differentiation through Acvr1b‐mediated TGF‐β/Smad3 signaling. Key findings reveal that overloading shifts chondrocytes from a proliferative (Col1a1/Sox9‐expressing) to hypertrophic (Col10a1/Runx2‐expressing) state, with pseudotime trajectory and CytoTRACE analyses confirming reduced stemness and irreversible differentiation progression.

Central to our study process is the identification of Acvr1b as a critical regulator validated both in vivo and in vitro: its upregulation under overloading correlates with heightened TGF‐β/Smad3 activity and terminal differentiation markers, while siRNA‐mediated Acvr1b inhibition restores chondrocyte proliferation and attenuates hypertrophic gene expression.

Previous research reports that Acvr1b is crucial in regulating the proliferation of hematopoietic cells, neuronal differentiation, pituitary hormone secretion, and tissue repair, primarily through the Smad2 and Smad3 proteins [17, 18]. It shares the same signaling pathway with TGF‐β at the level of Smad2 and Smad3 [19, 20]. Several studies have highlighted the significant role of Acvr1b in regulating the differentiation of various cell types. Yuki Akimoto et al. demonstrated that Acvr1b critically regulates oocyte maturation through its dual roles in mitotic proliferation and meiotic differentiation [14]. Yosuke Mizuno et al. demonstrated in vitro that Acvr1b regulates osteogenic differentiation of ST2 stromal cells derived from bone marrow [15]. Other researchers suggest that Acvr1b also plays a critical role in cardiomyocyte fibrosis induced by angiotensin II (Ang II) [21]. Our research fills the blank of acvr1b regulating terminal differentiation in the TMJ.

The TGF‐β signaling pathway serves as a critical regulator of diverse cellular processes, orchestrating proliferation, differentiation, apoptosis, and homeostatic maintenance across both embryonic development and adult tissue physiology [22]. TGF‐β can promote angiogenesis, infiltrate subchondral bone, exacerbate osteoarthritis progression, and increase the expression of terminal differentiation genes such as Mmp13, Runx2, and Col10a1 [23]. Michèle M. G. Hillege et al. found that Acvr1b can stimulate muscle fiber hypertrophy and promote muscle regeneration via the TGF‐β pathway, indicating a close relationship between Acvr1b, Smad2, Smad3, and TGF‐β in regulating cell differentiation [24]. These results increase the feasibility of acvr1b regulating cartilage terminal differentiation through TGF ‐ *β* under overloading. Our study aligns with prior reports implicating TGF‐β signaling in cartilage homeostasis but diverges by pinpointing Acvr1b—rather than canonical TGF‐β receptors—as the dominant mechanoresponsive driver in TMJ degeneration, a novel mechanistic insight.

The clinical relevance of these findings is underscored by the absence of disease‐modifying therapies for TMJ degeneration. Our data suggest that targeting Acvr1b could mitigate pathological chondrocyte maturation, offering a precision strategy to preserve cartilage integrity. However, there still some challenges remain. While our murine model replicates key features of human TMJ overloading, interspecies differences in cartilage repair capacity and loading mechanics necessitate validation in human‐derived chondrocytes or explant models. Furthermore, A notable limitation of this study is its current focus on cellular and transcriptomic alterations without accompanying protein‐level validation or in vivo mechanistic confirmation. To address this gap, future work will employ Cre‐loxP mouse models to validate Acvr1b′s role in chondrocyte terminal differentiation and cartilage degeneration under overloading, thereby bridging molecular insights to functional pathophysiology.

Despite these constraints, our multi‐modal approach—combining snRNA‐seq, pseudotime analysis, and functional validation—provides a robust framework for exploring mechanotransduction pathways in cartilage disorders. This study preliminarily validates the mechanism by which Acvr1b regulates chondrocyte terminal differentiation and offers a novel therapeutic strategy for precise and efficient intervention in terminal chondrocyte differentiation under overloading conditions.

## 4 Methods

According to Fang et al. [7], after 20 days of continuous mechanical load, TMJ degenerative damage does not recover spontaneously. Therefore, we divided the male mice into two groups: control and overloading. After anesthesia, we measured the maximum mouth opening and applied a large‐opening device made from 18 × 25 stainless steel square wire. The force was set to 100g using an orthodontic dynamometer. The control group was only anesthetized, and no further treatment was applied. After 8 weeks of growth, the experiment was conducted with continuous treatment applied for 20 days, 1 h per day. On the 20th day, the mice were sacrificed, and bilateral condylar cartilage was collected for single‐nucleus cell analysis.

### 4.1 Nuclei Isolation Sorting From Mice Condylar Cartilage Tissues

Male mice's condylar cartilage tissues were removed and then cleaned in PBSE (PBS buffer with 2 mM EGTA) that had been chilled beforehand. In accordance with the manufacturer's product handbook, GEXSCOPE Nucleus Separation Solution (Singleron Biotechnologies, Nanjing, China) was used to isolate the nuclei. Trypan blue was used to count the isolated nuclei after they were resuspended in PBSE to 106 nuclei per 400 μL and passed through a 40 μm cell strainer. DAPI (1:1,000) was used to stain PBSE‐enriched nuclei (TermoFisher Scientific, D1306). Singlets that were DAPI‐positive were defined as nuclei.

### 4.2 Pathway Enrichment Analysis

Functional pathway analysis was conducted using the Kyoto Encyclopedia of Genes and Genomes (KEGG) database via the clusterProfiler R package (v3.16.1) [25]. Significantly enriched pathways were identified using an adjusted *p*‐value threshold of adjusted *p* < 0.05. Key pathways were visualized using bar plots to highlight their biological relevance.

### 4.3 Cell‐Type Recognition With Cell‐ID

Cell‐ID is a multivariate method that uses hypergeometric tests (HGT) to identify individual cells by extracting their gene signatures. The most highlighted gene sets of each cell were then determined by calculating a gene ranking for each cell. Since the cell type with the lowest HGT *p* value was identified, each cell's identity was ascertained. The frequency of each cell type within each cluster was determined for cluster annotation, and the cell type with the greatest frequency was selected as the cluster's identification.

### 4.4 Subtyping of Major Cell Types

To enable higher‐resolution analysis of chondrocyte heterogeneity, cells from the target cluster were isolated, re‐clustered using the previously described pipeline, and partitioned into six distinct subpopulations (chondrocyte subclusters 1–6). This subclustering approach generated a refined cellular map of chondrocyte states.

### 4.5 Cell‐Cell Interaction Analysis (CellChat)

The scRNA‐seq data's intercellular communication networks were examined using CellChat (version 0.0.2). The R package was used to construct a CellChat object. The object's metaslot was updated with cell information. The matching receptor inference computation was carried out once the ligand‐receptor interaction database was established.

### 4.6 Cell‐Cell Interaction Analysis (CellCall)

CellCall v0.0.0.9000 [26] was employed to infer intrinsic regulatory signaling pathways by evaluating intercellular communication through receptor‐ligand interactions between cell types/subtypes. The interaction strength was quantified using the L2 norm of ligand‐receptor pairs combined with downstream transcription factor (TF) activity scores derived from the built‐in GSEA method. This approach enabled the assessment of the proportion of ligand‐receptor gene connections across cell populations. Significant ligand‐receptor‐TF interactions (*p* < 0.05, hypergeometric test) were identified and visualized using CellCall's integrated plotting functions.

### 4.7 Cell Differentiation Potential Evaluation: CytoTRACE

The differentiation potential of cell subpopulations was predicted using CytoTRACE (v0.3.3) [27], a computational tool that infers cellular differentiation status from single‐cell RNA sequencing (scRNA‐seq) data based on gene counts and expression profiles.

### 4.8 Pseudotime Trajectory Analysis: Monocle2

Using Monocle2 v 2.10.0 [28], the differentiation trajectory of monocyte subsets was reconstructed using single‐cell RNA sequencing data. Highly variable genes (top 2000) were selected via Seurat (v3.1.2, FindVariableFeatures) for pseudotemporal ordering. DDRTree was applied for dimensionality reduction, and trajectory visualization was generated using the plot_cell_trajectory function in Monocle2.

### 4.9 HE Staining

Tissues from the temporomandibular joint (TMJ) were collected and preserved in 10% neutral buffered formalin. Sagittal slices were then cut from the paraffin‐embedded fixed tissues. A succession of ethanol solutions was used to rehydrate these sections after they had been deparaffinized in xylene. Hematoxylin was used for nuclear staining and eosin for cytoplasmic staining after the slices had been rehydrated. Following another round of ethanol and xylene dehydration, the stained sections were placed on a coverslip for microscopic inspection.

### 4.10 SA‐β‐Gal Staining

In order to replicate overloaded situations, chondrocytes were cultivated and exposed to various centrifugal forces. Following treatment, cells were fixed in 4% paraformaldehyde (PFA) for 15 min at room temperature (RT). After fixation, they were incubated with SA‐β‐Gal staining solution at 37°C for 12–16 h, then washed three times with phosphate‐buffered saline (PBS). SA‐β‐Gal‐positive cells were quantified by manual counting under bright‐field microscopy.

### 4.11 Safranin O Staining

Safranin O‐fast green was used to prepare and stain sagittal slices of the TMJ. The color variations between the subchondral bone, which was stained green, and the condylar cartilage, which appeared red, were noted when the sections were examined and recorded.

### 4.12 CCK‐8 Assay

Cells of condylar cartilage were cultivated in both controlled and overloaded environments. After adding CCK‐8 solution to the growth media at the 120‐ and 168‐h time periods, the cells were incubated for two to four hours at 37°C. A microplate reader was used to measure the absorbance at 450 nm, and the results were examined to compare the growth rates of the overloaded and control groups.

### 4.13 SiRNA Knockdown

We developed and manufactured siRNAs that target Acvr1b (siRNA‐180, siRNA‐925) as well as a non‐targeting control siRNA (siRNA‐NC). Using an appropriate transfection reagent, the siRNAs were transfected into condylar cartilage cells. Total RNA was isolated from the cells after they had been collected for 48 h. Acvr1b expression levels were measured by qPCR, and the results were examined to assess the effectiveness of siRNA knockdown.

### 4.14 TUNEL Staining

TUNEL labeling was carried out in accordance with the manufacturer's procedure on tissue slices taken from the TMJ samples in order to identify apoptotic cells. Cell nuclei were seen by counterstaining the slices with DAPI. Observations and documentation of the staining findings revealed the existence of cells that were TUNEL‐positive. The proportion of cells that were TUNEL‐positive to all cells was measured.

### 4.15 EdU Staining

Mice were given EdU (10 μL/g) intraperitoneally 24 h before they were put to sleep. Sections of the condylar cartilage were prepared after it was harvested. To identify proliferating cells, EdU staining was carried out in accordance with the manufacturer's instructions. To see the cell nuclei, DAPI was used as a counterstain on the sections. The location of EdU‐positive cells was noted in the staining findings, which were observed and recorded. In the proliferative and hypertrophic zones, the proportion of EdU‐positive cells was measured.

### 4.16 Statistical Analysis

All data were analyzed using GraphPad Prism 9.4.1 (GraphPad Software, San Diego, CA). Comparisons between two groups were performed using two‐tailed unpaired Student's *t*‐tests, while one‐way ANOVA was used for multi‐group comparisons. Data are presented as mean ± SEM. Statistical significance was defined as *p* < 0.05 (*p < 0.05, **p < 0.01, ***p < 0.001), with “NS.” (not significant) indicating no statistically significant difference.

## Author Contributions

D.Z., Y.Z., Z.T. and O.L. conceived and designed the study. D.Z. performed most of the experiments. Y.J., H.Z. and X.L. contributed to some experiments. D.Z., Y.H., X.W. and Y.L.(Liang) wrote the manuscript. Y.Z., Z.T., O.L., V.P. and Y.L.(Li) oversaw all experimental design, data analysis, and manuscript preparation. All authors have read and agreed to the published version of the manuscript.

## Ethics Statement

The animal experiments in this study were approved by the Animal Ethics Committee of Xiangya Stomatological Hospital, Xiangya School of Stomatology, Central South University (number: 20210087). All efforts were made to minimize suffering.

## Conflicts of Interest

The authors declare no conflicts of interest.

## Supporting information

Supporting Information S1
