# Supplementary material for: Mechanistic Insights Into Overloading‐Induced Terminal Differentiation of TMJ Condylar Cartilage at the Single Cell Level
Source: Smart Med. 2025 Jul 30;4(3):e70011. doi: 10.1002/smmd.70011 (PMC12362756; doi:10.1002/smmd.70011)
Supplement: Supplementary file 1 — Supporting Information S1 [file SMMD-4-e70011-s001.docx]

**Supplementary**

Micro-computed tomography (micro-CT) three-dimensional reconstructions were performed on the TMJ of control and overloading groups. The superior view of the reconstructions revealed an increased number of concave areas in the condylar bone tissue of mice subjected to overloading, indicating bone tissue destruction. This trend was observed in both male and female mice. Additionally, sagittal cross-sectional scans showed a widening of the trabecular bone space in the subchondral bone of both sexes, along with an increase in bone cavity volume and a reduction in the thickness of the superficial cartilage layer (Supplementary A).

We quantitatively assessed subchondral bone architecture in control and mechanically overloaded mice, comparing sex-specific differences in key microstructural parameters: bone volume fraction (BV/TV), trabecular number (Tb.N), trabecular thickness (Tb.Th), and trabecular separation (Tb.Sp). After overloading, we found significant reductions in BV/TV and Tb.Th, and an increase in Tb.Sp (P < 0.05). No significant difference in Tb.N was observed between the groups (P > 0.05). Notably, male mice showed more pronounced changes in subchondral bone parameters, suggesting that they are more susceptible to the effects of mechanical stimulation (Supplementary B). Based on these findings, male mice were selected for single-nucleus cell sequencing.

| 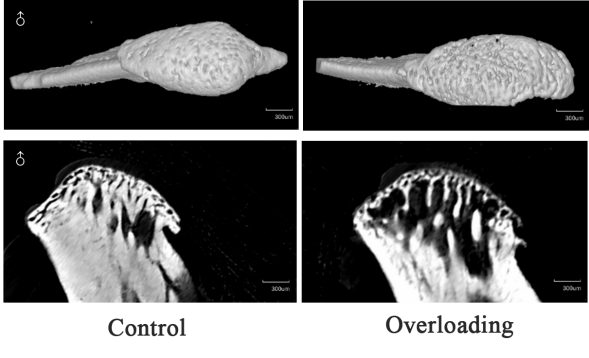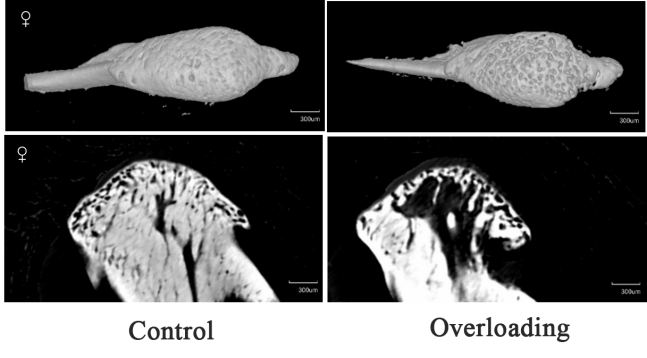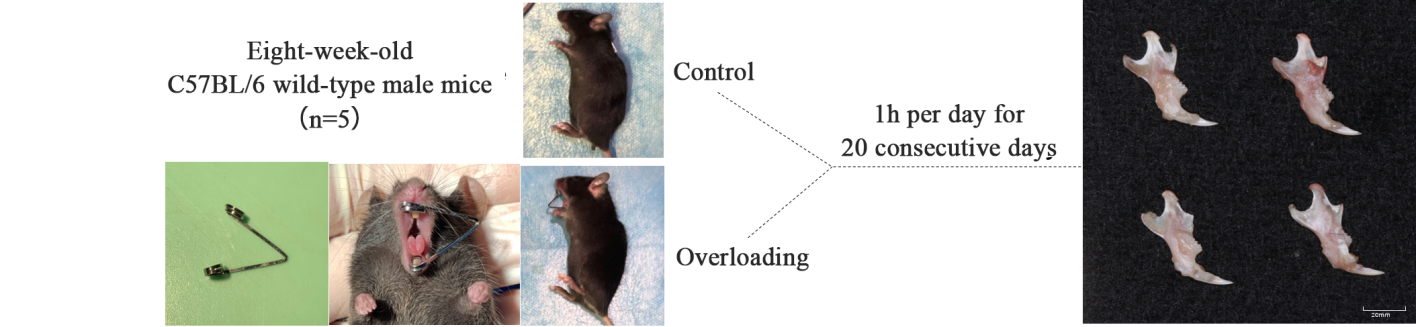  **B**  **A** |
| --- |

**Supplementary.** (A) Schematic diagram of the construction of the overloaded force mouse model.

(B) Comparative micro-CT analysis of the temporomandibular joint (TMJ) in the Overloading group and the control group mice. All data were shown as means ±SEM. *, *p* < 0.05; **, *p* < 0.01; ***, *p* < 0.001; NS., *p* > 0.05.
